# Author Correction: Markerless tracking of an entire honey bee colony

**DOI:** 10.1038/s41467-021-23297-4

**Published:** 2021-05-19

**Authors:** Katarzyna Bozek, Laetitia Hebert, Yoann Portugal, Alexander S. Mikheyev, Greg J. Stephens

**Affiliations:** 1 Biological Physics Theory Unit, OIST Graduate University, Okinawa, Japan; 2 Ecology and Evolution Unit, OIST Graduate University, Okinawa, Japan; 3 Present Address: Research School of Biology, Australian National University, Canberra, Australia; 4 Department of Physics and Astronomy, Vrije Universiteit Amsterdam, Amsterdam, The Netherlands; 5 University of Cologne, Center for Molecular Medicine Cologne (CMMC), Faculty of Medicine and University Hospital Cologne, Cologne, Germany

**Keywords:** Image processing, Biological physics

Correction to: *Nature Communications* 10.1038/s41467-021-21769-1, published online 19 March 2021.

The original version of this Article omitted from the author list the fourth author Alexander S. Mikheyev, who is from the Ecology and Evolution Unit, OIST Graduate University, Okinawa, Japan, and the Research School of Biology, Australian National University, Canberra, Australia. The third author Yoann Portugal has the following additional affiliation: Ecology and Evolution Unit, OIST Graduate University, Okinawa, Japan. The fourth author Alexander S. Mikheyev and the fifth author Greg J. Stephens declare equal contributions. Consequently, the Acknowledgements, which formerly read “We thank Michael Iuzzolino, Dieu My thanh Nguyen, Orit Peleg, and Michael Smith for comments on the manuscript and code testing. This work was supported by the Okinawa Institute of Science and Technology Graduate University”, have been corrected to “We are grateful to Takahashi Ikemiya for maintaining the experimental bee colonies. We thank Michael Iuzzolino, Dieu My Thanh Nguyen, Orit Peleg, and Michael Smith for comments on the manuscript and code testing. This work was supported by the Okinawa Institute of Science and Technology Graduate University. Additional funding was provided by KAKENHI grants 16H06209 and 16KK0175 from the Japan Society for the Promotion of Science to AM”. Additionally, the Author Contributions, which formerly read “Y.P. performed the bee work and devised the imaging setup, L.H. devised the labeling tool, K.B. performed method development and data analysis, K.B. and G.S. designed the study and wrote the manuscript”, has been corrected to “Y.P. performed the bee work, Y.P. and A.M. devised the imaging setup, L.H. devised the labeling tool, K.B. performed method development and data analysis, K.B., A.M., and G.S. designed the study, K.B. and G.S. wrote the manuscript”. This has been corrected in both the PDF and HTML versions of the Article.

The original version of the Supplementary information associated with this Article contained an error in the description of Supplementary Table 2, which incorrectly read “All imaging data in this study were collected in 2019”. The correct version states “2018” in place of “2019”. The HTML has been updated to include a corrected version of the Supplementary information.

## Supplementary information

Supplementary Information

