# Supplementary material for: Markerless tracking of an entire honey bee colony
Source: Nat Commun. 2021 Mar 19;12:1733. doi: 10.1038/s41467-021-21769-1 (PMC7979864; doi:10.1038/s41467-021-21769-1)
Supplement: Supplementary file 1 — Supplementary Information [file 41467_2021_21769_MOESM1_ESM.pdf]

Markerless tracking of an entire honey bee colony

Supplemental Material

1. Figures

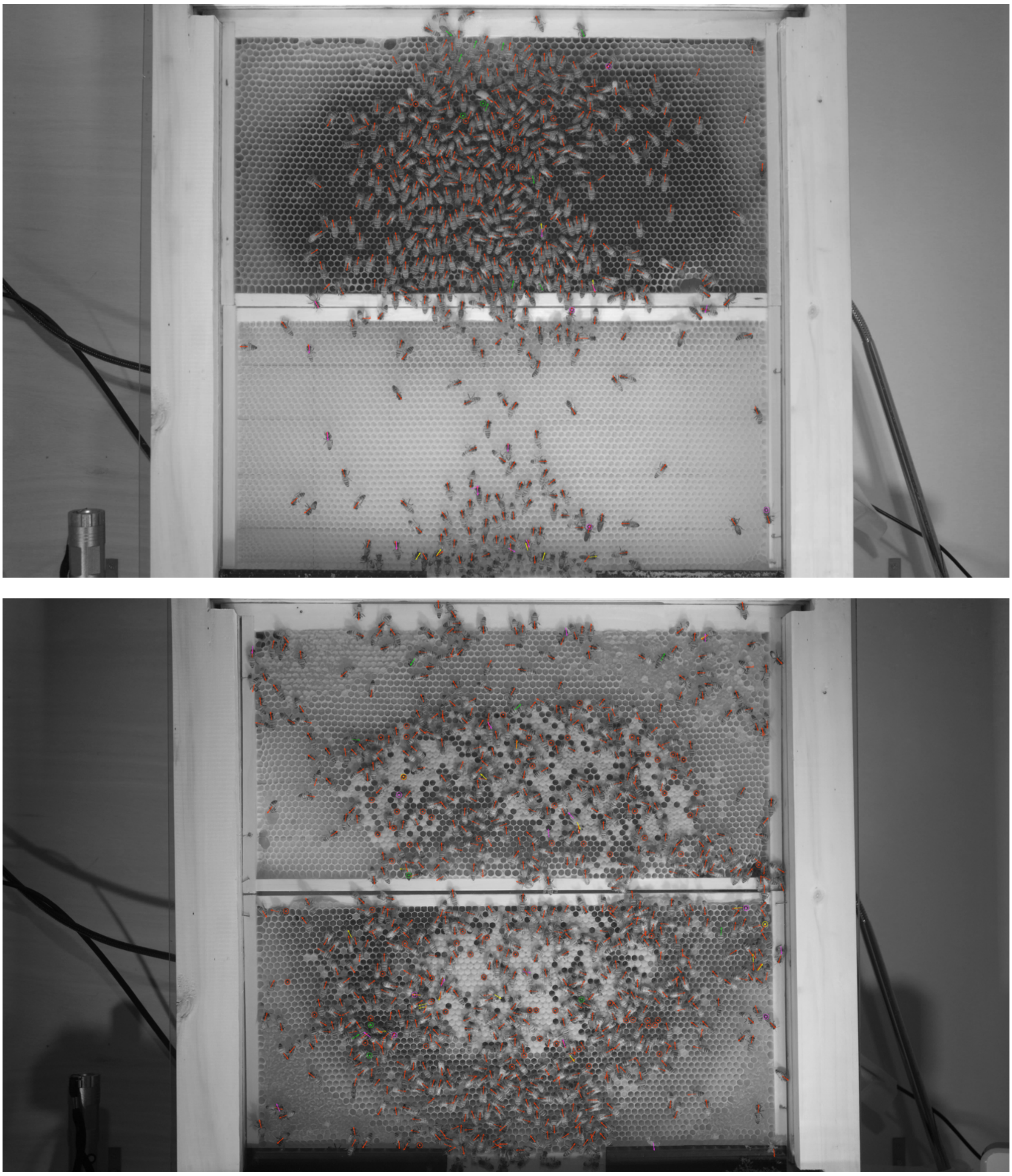

Supplementary Figure 1. Detection accuracy in recordings L5 (upper panel) and S5 (bottom panel). Each detection's center is marked with a dot. Detections recognized as cell-bees are marked with round symbols, full-bees are marked with arrows that indicate the bee head-tail orientation angle. These two recordings were not part of the training set for the detection and tracking methods. Yellow color indicates labels, overlapping red color true positive predictions. False positives are marked in magenta, false negatives in green.

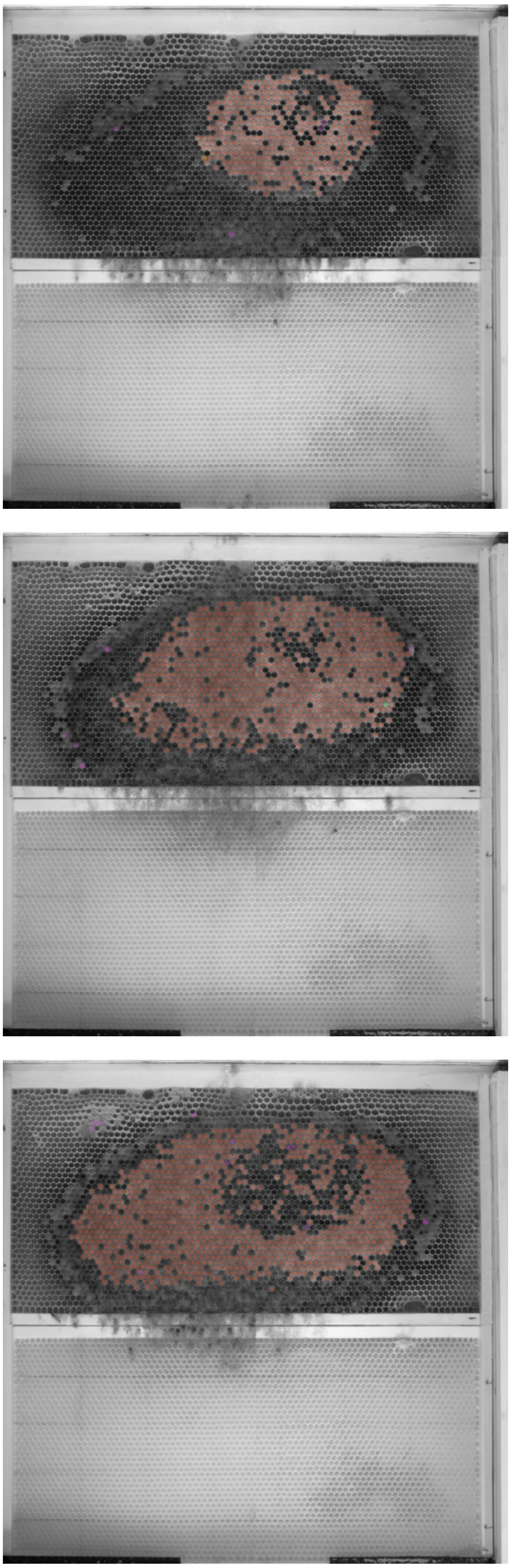

Supplementary Figure 2. Example of brood cell labeling and detection in video L5. This video was not part of the train set, the coloring is analogous to the image S1 above.

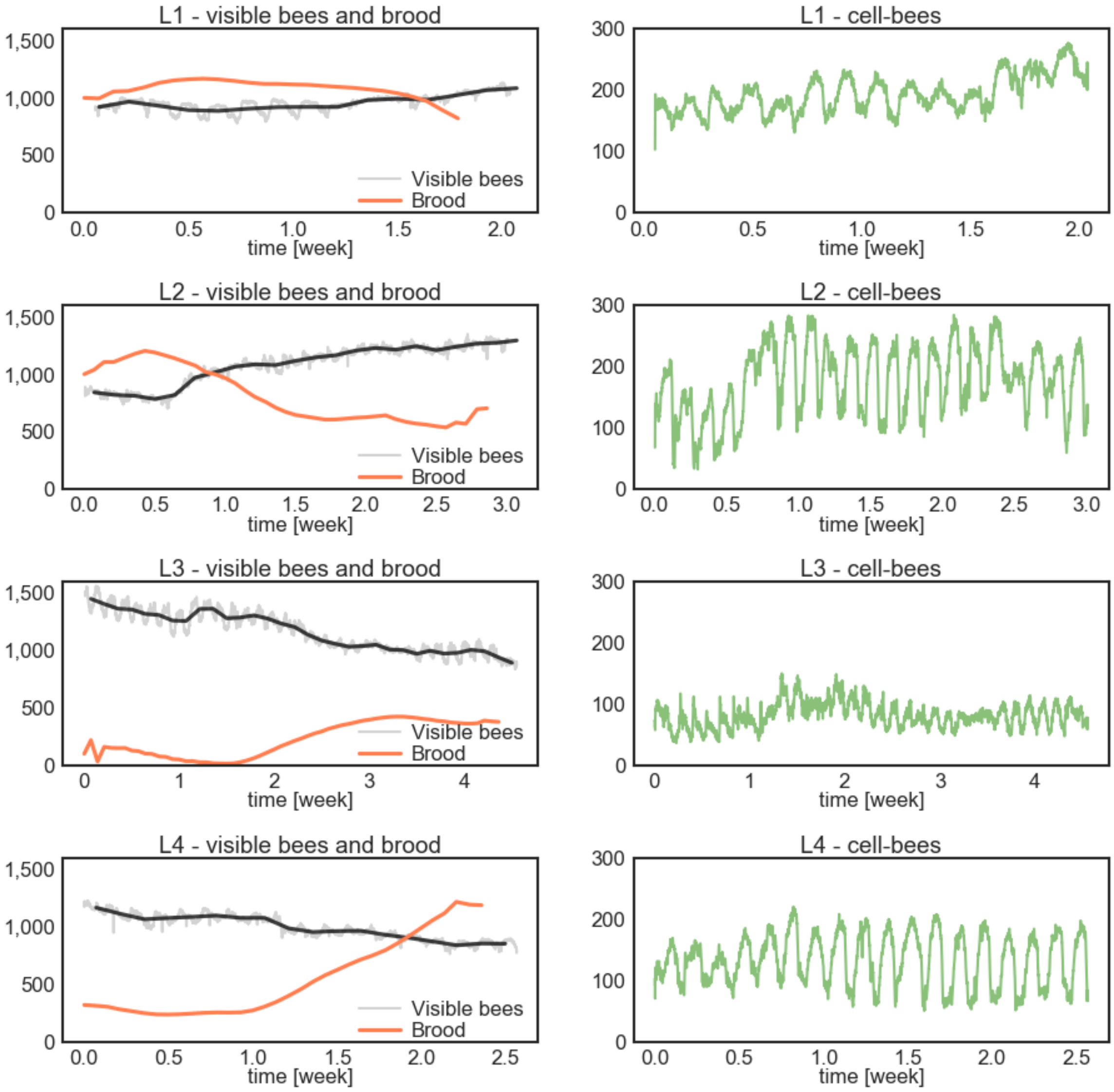

Supplementary Figure 3. Visible bee, brood, and cell-bee counts in recordings L1-L4. Y-axis represents counts of objects of each category indicated in the legend and plot title. Source data are provided as a Source Data file.

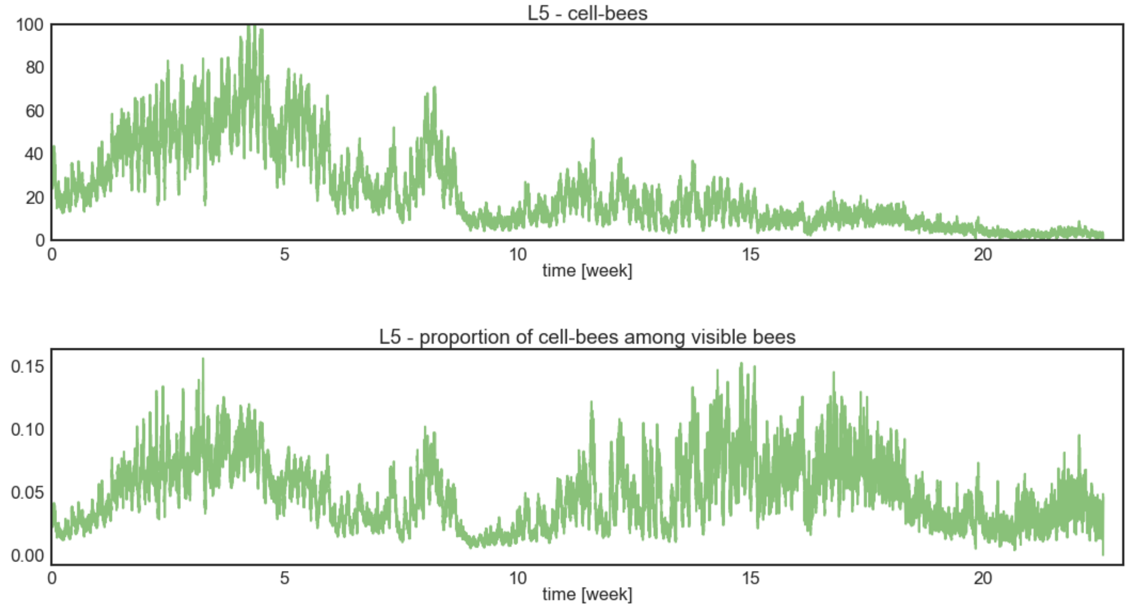

Supplementary Figure 4. Supplemental to Fig. 3c, brood cell, and cell-bee counts in beehive L5. Bottom panel shows numbers of cell-bees as a proportion of all bees detected in the hive in a given frame of the recording. Regardless of normalization method, the daily fluctuation of this count is present. Source data are provided as a Source Data file.

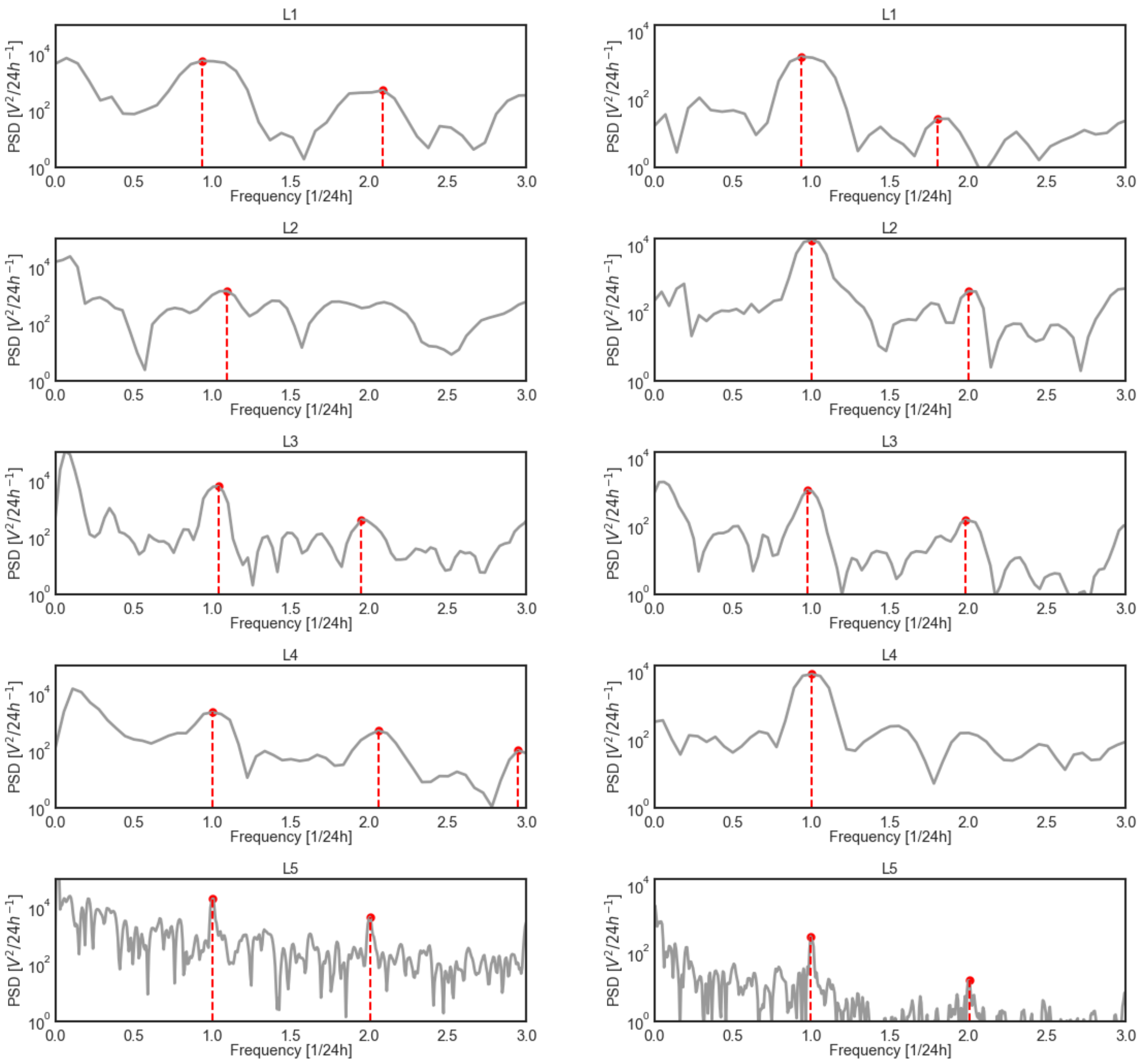

Supplementary Figure 5. Power spectrum density (PSD) of the population change (left panel) and cell-bee count (right panel), in recordings L1-L5. Local maxima, calculated over 5 h timespans are indicated with red dots in each plot. V stands for the time series variable – population and cell-bee count, respectively. Source data are provided as a Source Data file.

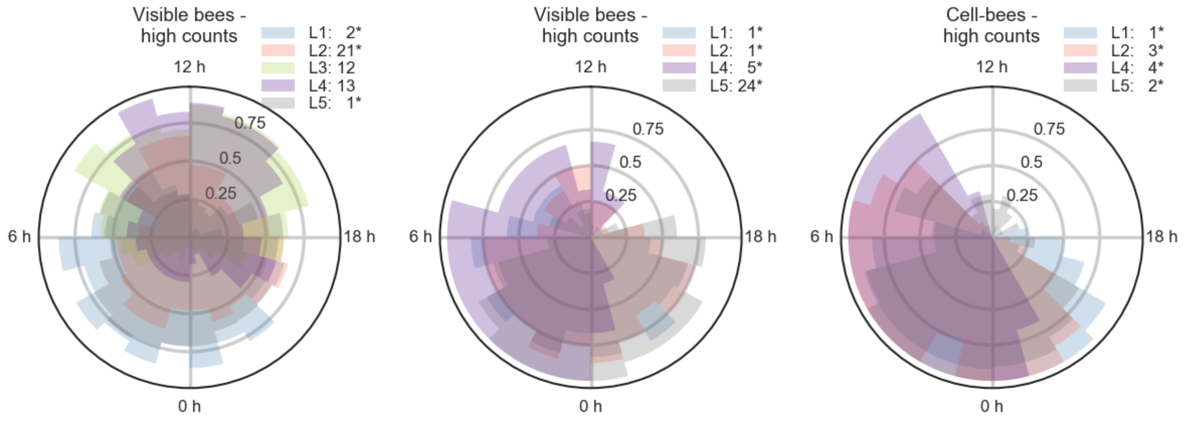

Supplementary Figure 6. Distributions of hours when the highest visible bee (left and middle panel) and cell-bee numbers (right plot) are present in hives L1-L5. Analogous to Fig. 3e, circular plots are separated into 24 bins. For each 24 h of a recording the number of times that the population or cell-bee counts are above the median number in the respective 24 h time window is counted in each 1 h bin and averaged over the number of days in the recording. Middle and right plots contain only days when number of brood cells in these hives exceeds 800. Numbers in the legend indicate mean time of the day when the high counts are observed. '*' symbol marks for hives where the observed high counts are not uniformly distributed based on $p < 0.0001$ in the Rayleight test of uniformity. While no clear pattern of the population fluctuation among the hives is present (left plot), on days with high brood count high numbers of bees are present predominantly at night which might reflect foraging activity in these hives (middle plot). On the same days the clear phase of cell-bees (right plot) is more pronounced compared to all recording days shown in Fig. 3e. Source data are provided as a Source Data file.

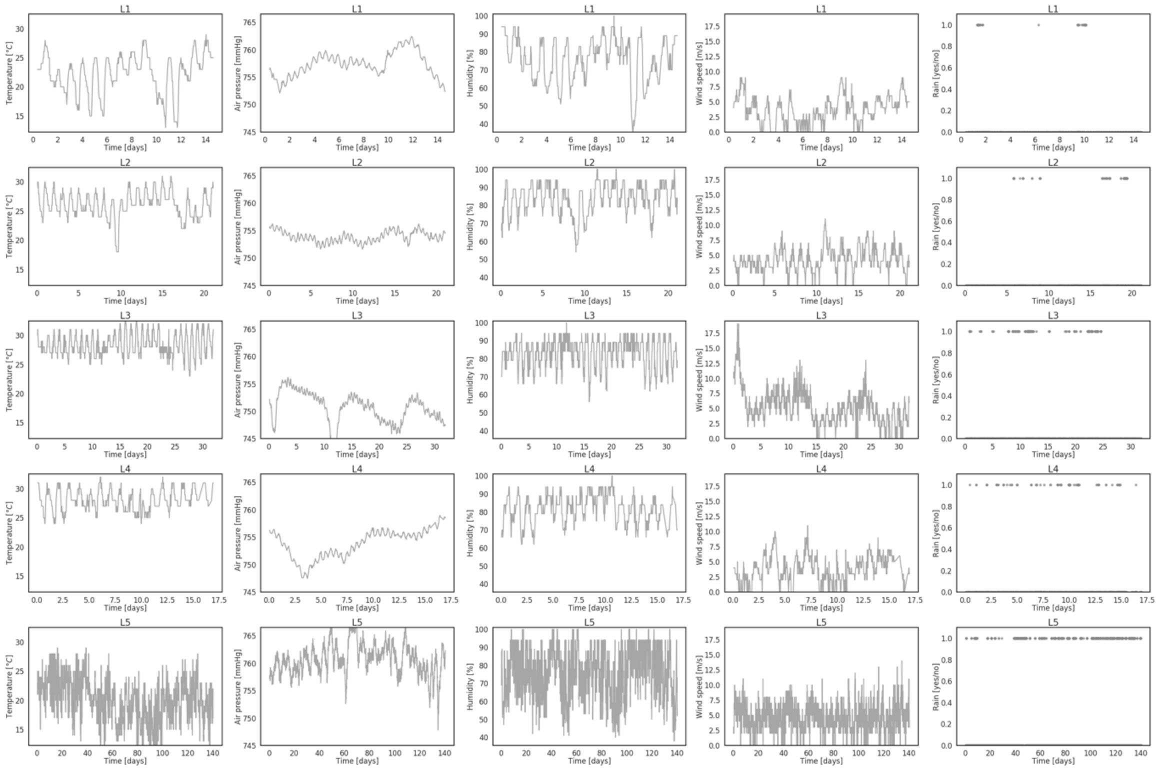

Supplementary Figure 7. Weather conditions during the time of recordings L1-L5. Shown are temperature, air pressure, humidity, wind, and rain reported in the location of the hives. While no extreme weather events were present during the recordings, hives L3 and L4 were recorded during particularly high temperatures which might be the reason for the anticorrelated phase of the population and cell-bee count. Source data are provided as a Source Data file.

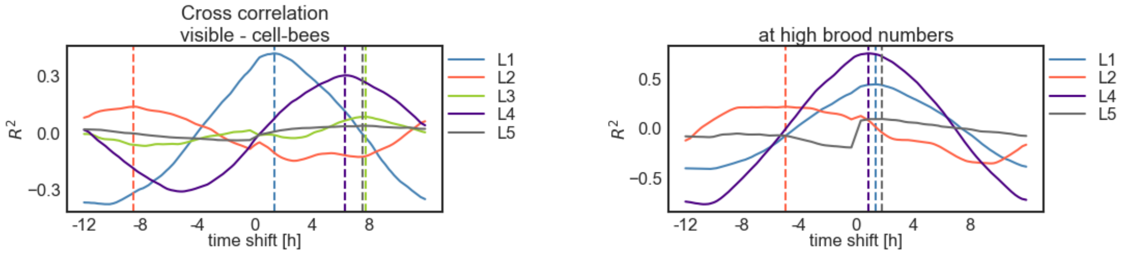

Supplementary Figure 8. Temporal cross-correlation between the total and cell-bee populations. Correlations between these two variables were calculated after shifting one of them (cell-bees) by an increasing number of 2-min-long offsets. We indicate in the plots the shift where the maximum correlation between the two variables is observed with a vertical dashed line. The plot on the right shows an analogous cross correlation analysis calculated on days of the recordings when the brood numbers are above 800. On those days the visible and cell-bee numbers are more closely in phase. Hive L3 did not show such high brood counts and was therefore not included in this panel. Source data are provided as a Source Data file.

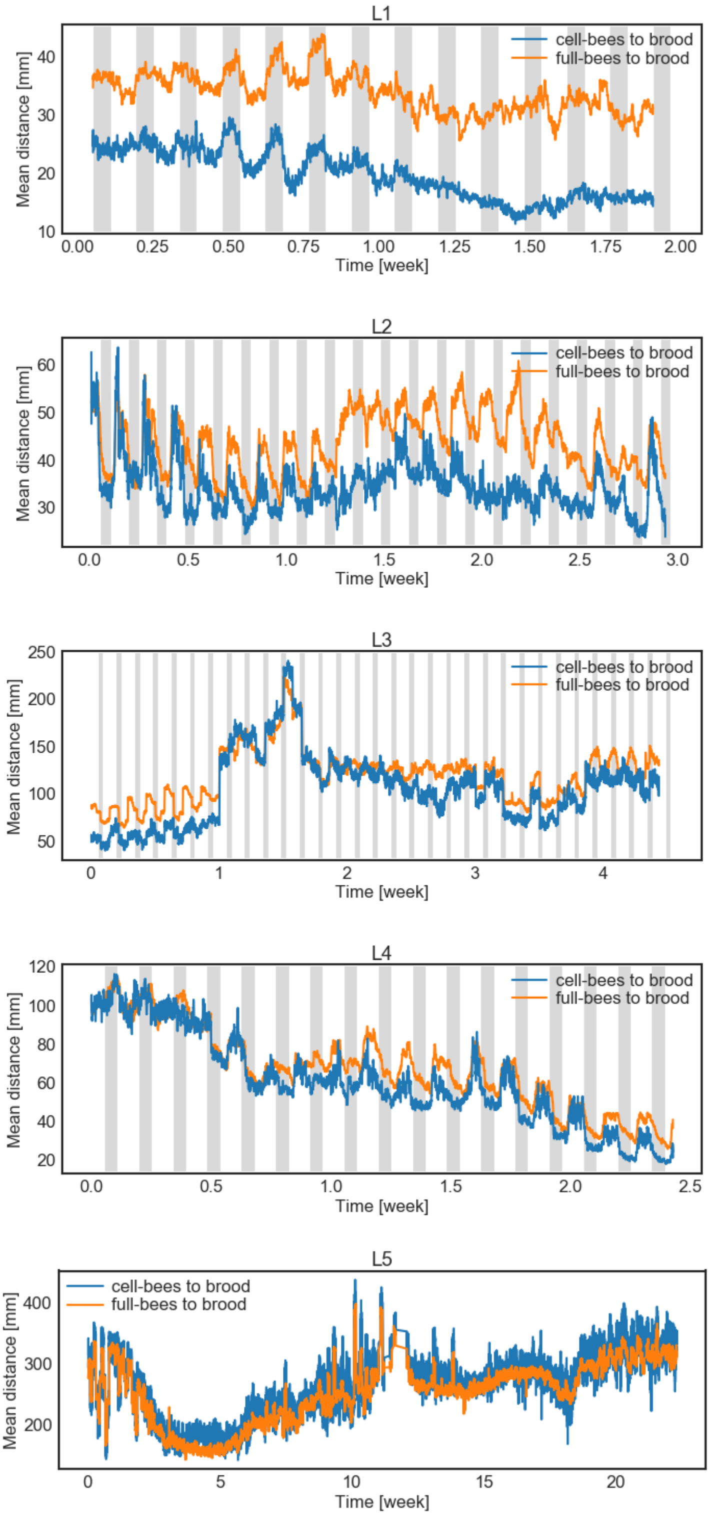

Supplementary Figure 9. Mean distance of each full-bee (blue) and each cell-bee (orange) to three closest brood cells. Both distances show daily fluctuations with peak times during the day suggesting that at night bees regroup around the brood. Bees inside comb cells tend to be closer to the brood cells suggesting the role of this activity in brood-related activities, such as thermoregulation. Shaded regions mark nighttime between 8pm and 8am. Due to the time span of L5 the shaded regions are not visible in this plot. Source data are provided as a Source Data file.

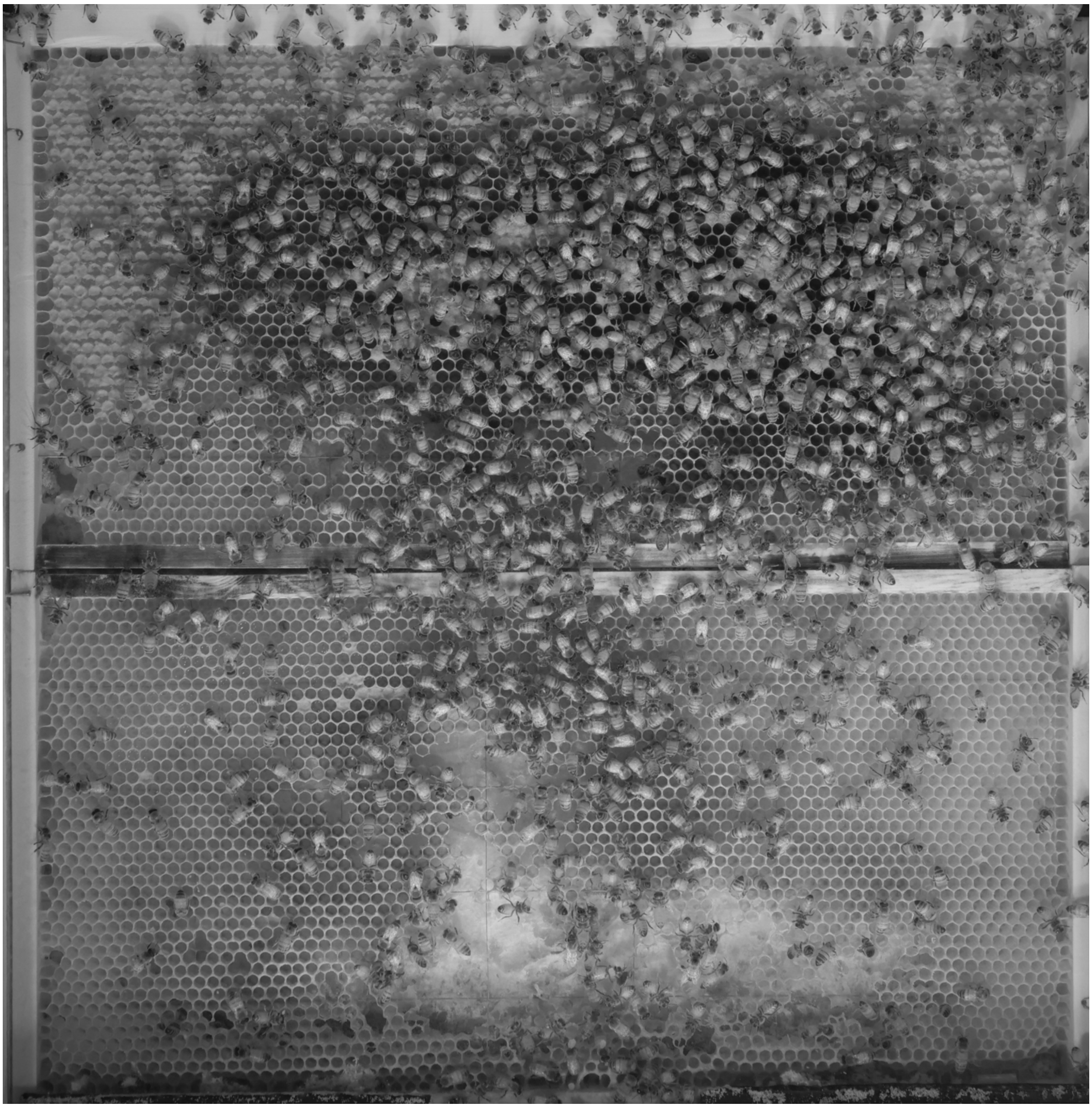

Supplementary Figure 10. Image of the hive L3 in the third week of the recording. Bottom frame was damaged due to moth infestation.

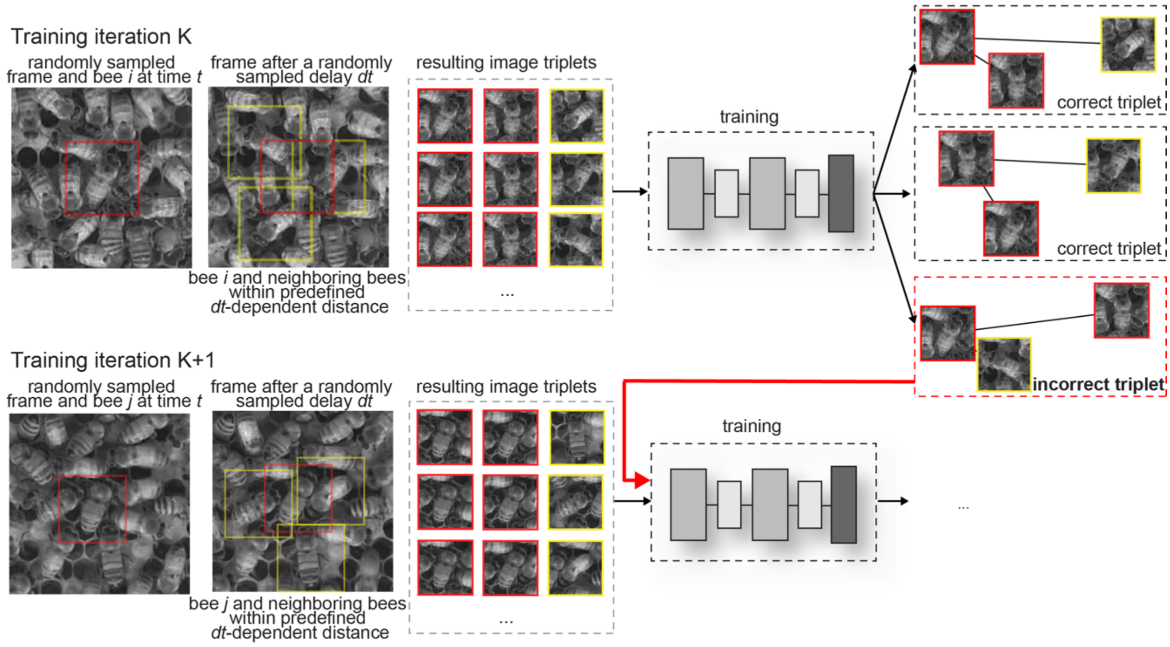

Supplementary Figure 11. Flow chart of the training procedure. In the following steps of the training not only randomly sampled triplets are used but also triplets that did not fulfill the embedding distance criteria in the preceding training step.

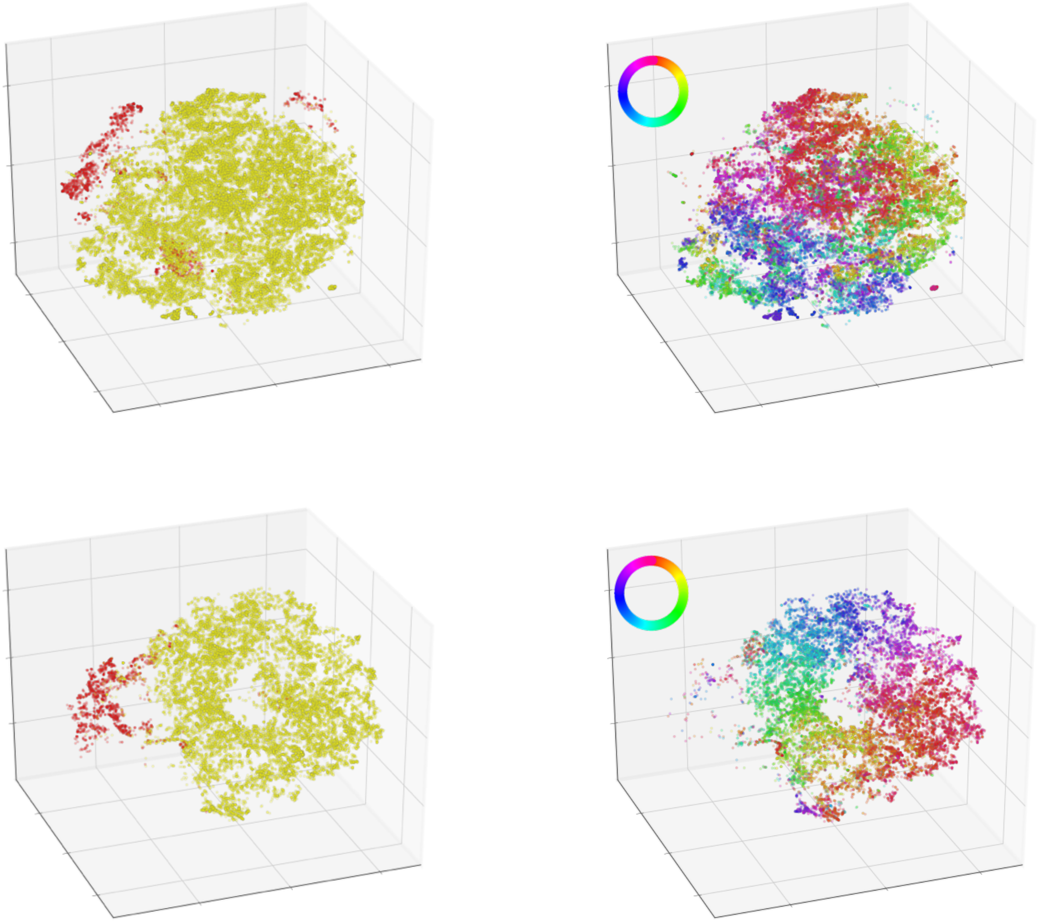

Supplementary Figure 12. Exploration of the embedding space – posture and orientation angle. Embeddings of all detections in Supplemental Movies M6 (upper plots) and M7 (lower plots) were transformed using t-SNE into 3d. Plots on the left are colored according to posture type with full-bees in yellow and cell-bees in red. Plots on the right are colored according to orientation angle as indicated in the circular legend. Both posture and orientation angle are important elements of the embeddings. Source data are provided as a Source Data file.

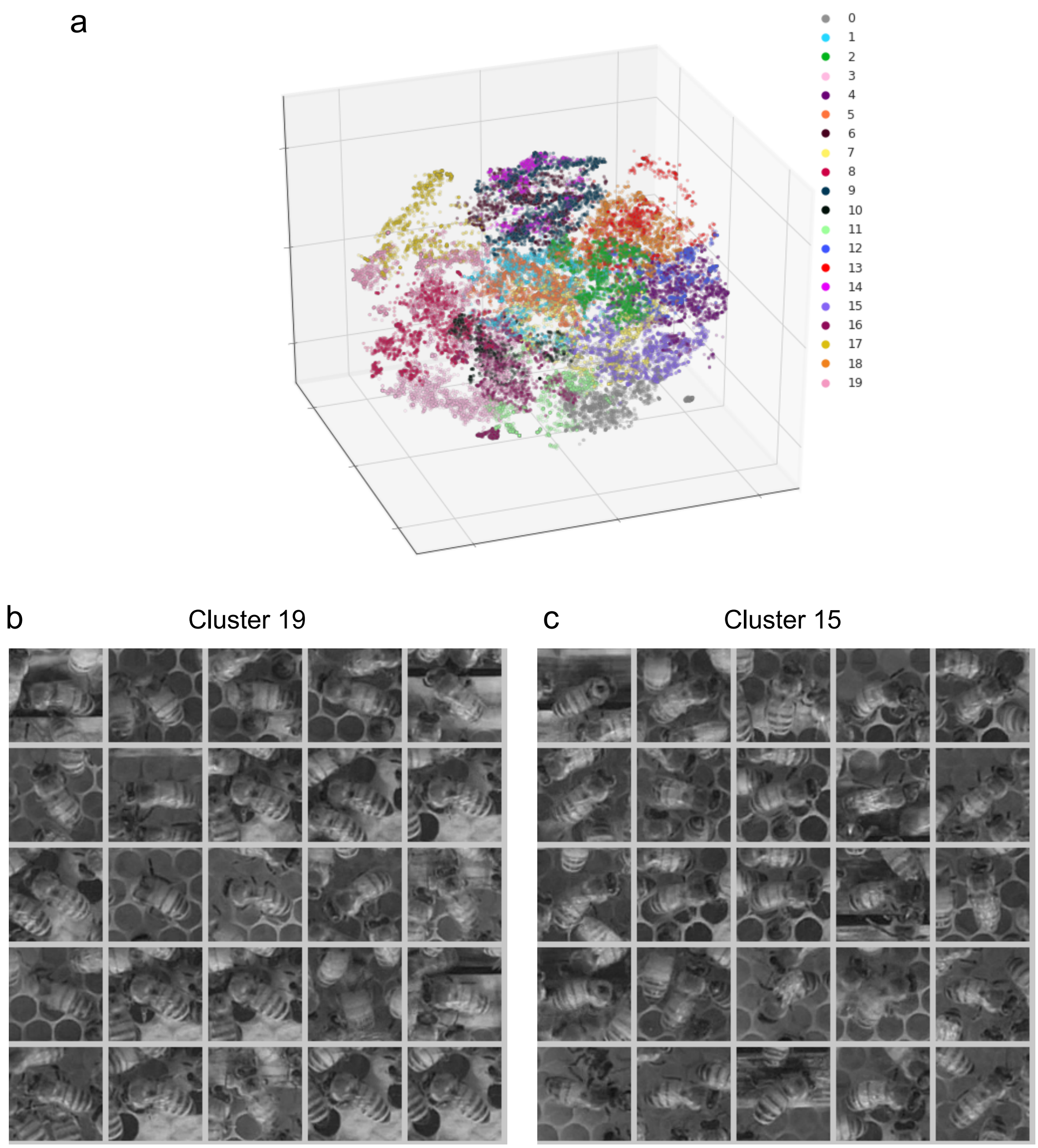

a
0
1
2
3
4
5
6
7
8
9
10
11
12
13
14
15
16
17
18
19
b
Cluster 19
c
Cluster 15

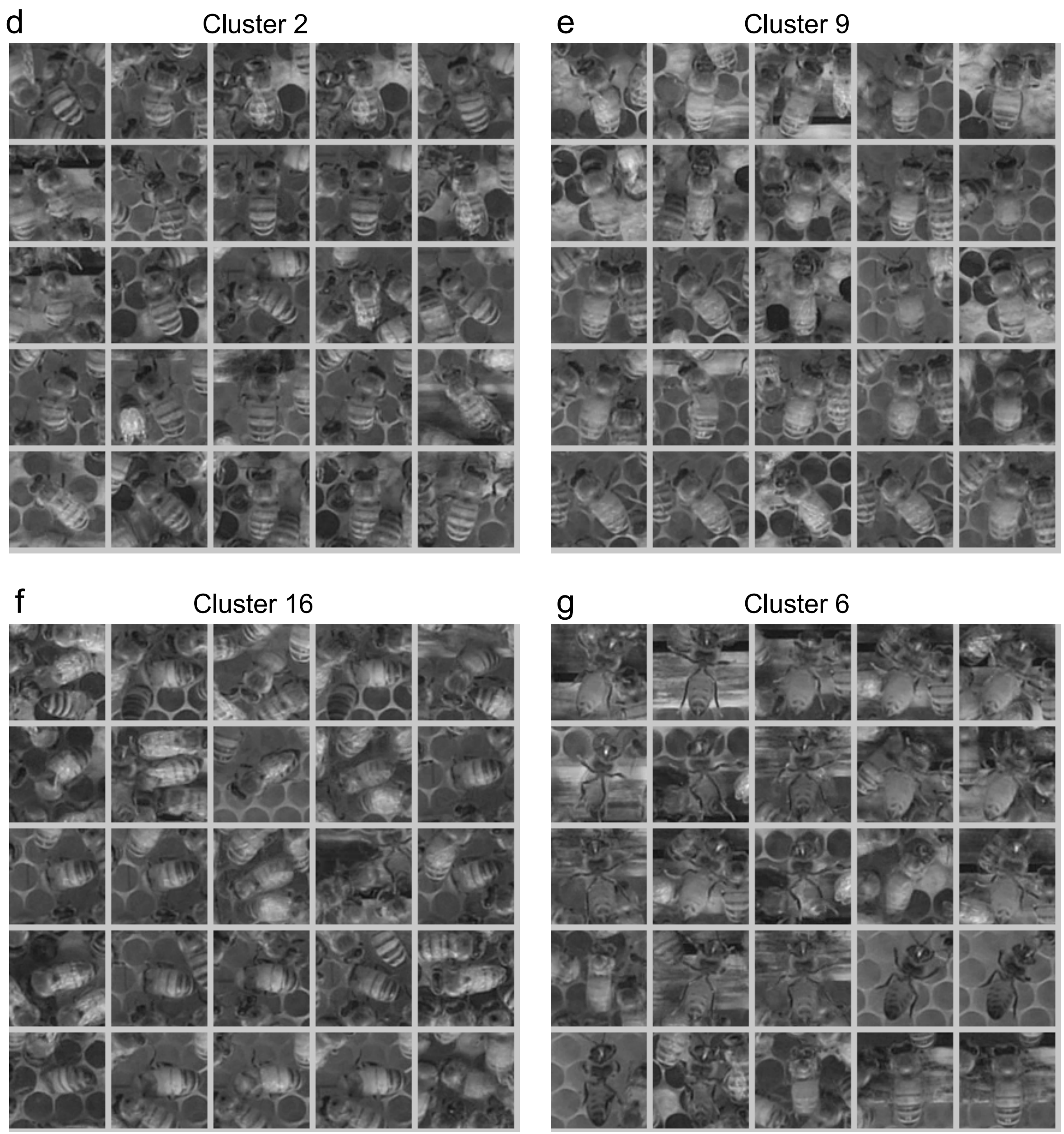

Supplementary Figure 13. Exploration of the embedding space. (a) Embedding space shown in Supplementary Figure 16 (upper panels) was split into 20 parts using KMeans clustering. Sets of images behind embeddings in each of the cluster are shown in panels b-g. (b-c) Orientation angle is an important element of the embeddings, bees in clusters 19 and 15 are oriented in opposing directions. (d-e) Background contribution to the embedding is exemplified in these two clusters. Even though both in cluster 2 and 9 the bees are oriented upwards, background of detections in these two differs with darker and more crowded background in cluster 2 and blighter and emptier background in cluster 9. (f-g) Posture is another component of the embeddings. Cluster 16 contains bees half hidden inside comb cells and pointing left. Cluster 6 contains bees walking on the glass of the beehive. All these visual features play important role in making correct matchings in the tracking procedure. Source data are provided as a Source Data file.

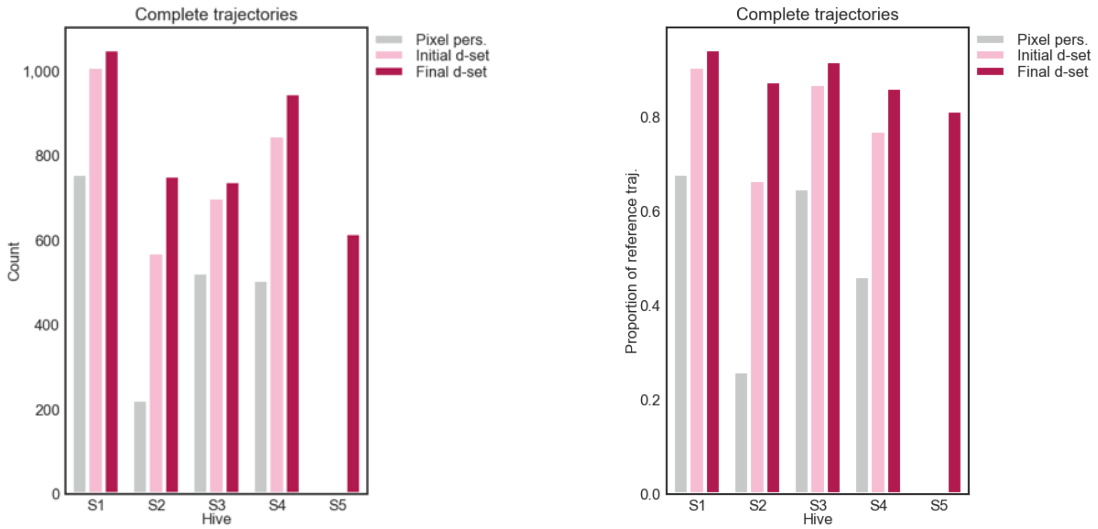

Supplementary Figure 14. Analogous to Fig. 4c – complete trajectories constructed with the respective methods and quantified as counts (left panel) and proportion of the number of all reference trajectories collected in beehives S1-S5.

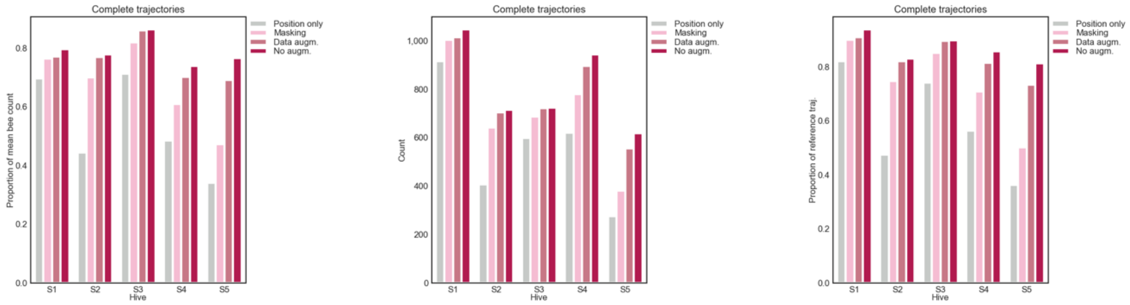

Supplementary Figure 15. Comparison of the number of correct trajectories constructed via position matching only and with embeddings derived from networks trained without and with augmentation and masking procedures. The numbers shown are relative to average number of detections in each respective hive (left), raw counts (middle), and relative to the number of reference trajectories in the respective hives (right). Using visual feature embeddings improves tracking, while masking and augmentation appear not to improve tracking results.

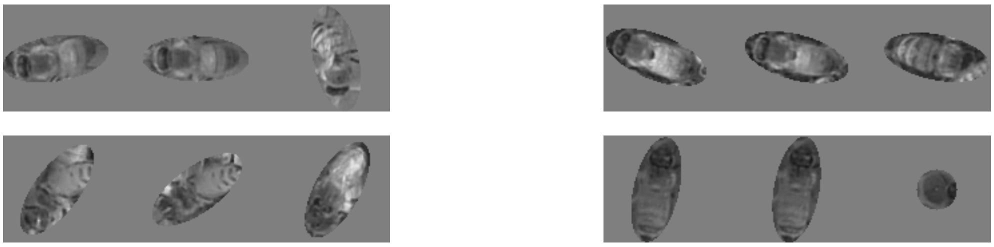

Supplementary Figure 16. Masking of background. To investigate the role of background in the accuracy of the matching procedure ellipse- and round-shaped masks were applied on the bee images during training and testing. Ellipse-shaped masks were applied to the full-bee images and rotated according to the bee body axis. Round-shaped masks were applied to the cell-bees. The images represent triplets of images that are fed into the network during training. From left to right each image contains anchor image, positive match, and negative match.

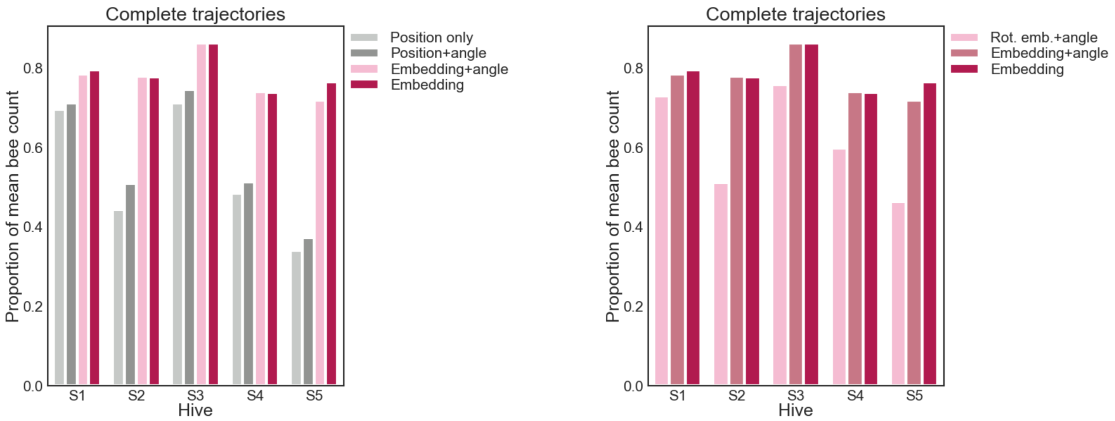

Supplementary Figure 17. Orientation angle in trajectory construction. Left plot: while adding orientation angle into the matching procedure does improve the "Position only" approach, it does not improve the approach based on embeddings of visual features. The reason for the lack of matching accuracy increase might be the fact that orientation angle is already encoded in the embedding. Right plot: disentangling contribution of the orientation angle to the embeddings of visual features is not straightforward. Approach "Rot. emb.+angle" uses for the matching visual embeddings of bees rotated to 0° orientation and the orientation angle. "Embedding+angle" and "Embedding" represent the same approaches as in the left plot. Adding angle information to embeddings of images from which the orientation information was removed ("Rot. emb.+angle") results in lower accuracy of matching than the "Embedding" only approach. Reasons for the decrease in performance might be in the complexity of the embeddings that combine orientation with posture and background composition information. Source data are provided as a Source Data file.

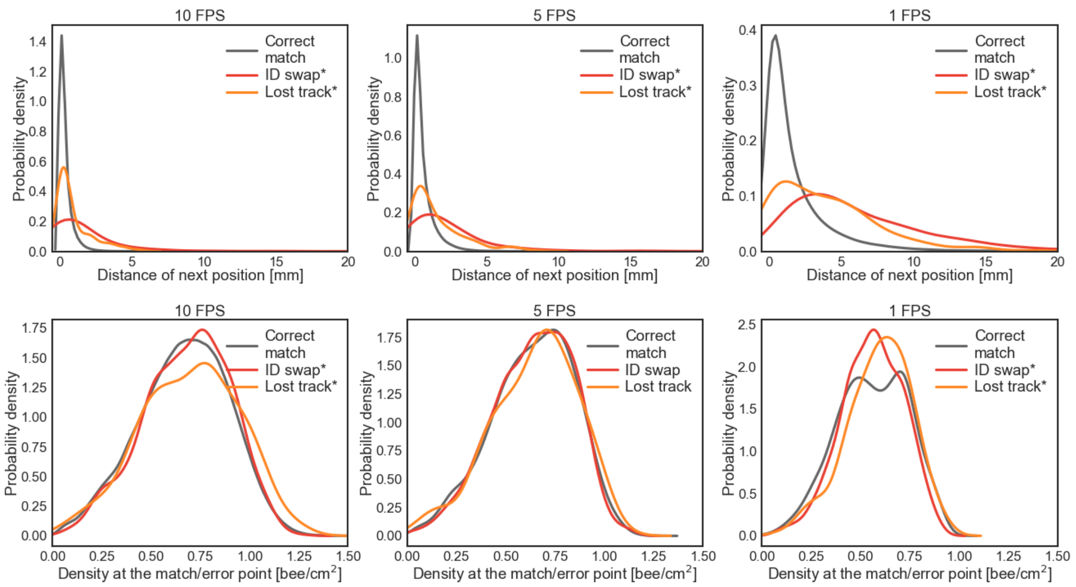

Supplementary Figure 18. Occurrence of errors with respect to distance of the next detection in a trajectory (upper panels) and bee density around the tracked bee (bottom panels). We counted identity swaps and lost trajectories (trajectories ending inside the hive before the end of recording) among trajectories matched at time resolutions of 10 FPS, 5 FPS, and 1 FPS. Significantly more identity swaps and lost trajectories occur as the distance of the next position in the trajectory increases. Bee density around the tracked bee shows less prominent nevertheless significant effect on the produced errors. Significant ($p < 0.001$, Kolmogorov-Smirnov test) differences with the distribution of the correct matches are indicated with '*' in the legend. Results are aggregated across recordings S1-S5 and obtained by comparison with the validated trajectories. Source data are provided as a Source Data file.

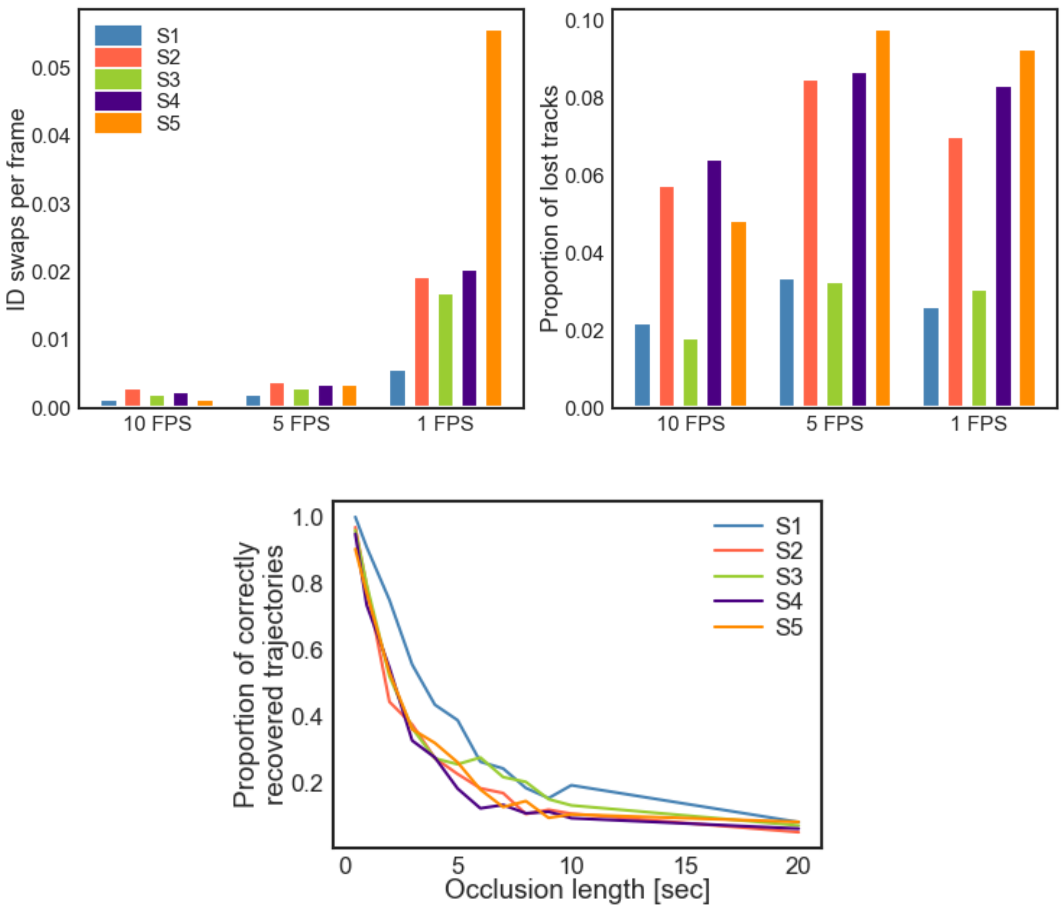

Supplementary Figure 19. Tracking errors at decreased frame rate and after occlusions. Numbers of identity swaps and lost trajectories at decreasing time resolution of the recordings are shown in the upper panels. Numbers of identity swaps (left panel) are relative to the number of bees and frames in a recording, numbers of lost trajectories (right panel) are relative to the average number of bees in the respective hives. Error numbers increase 0.3-2.5 times between 10 FPS and 5 FPS and 4-40 times between 10 FPS and 1 FPS. The bottom panel illustrates results of an occlusion test. 100 trajectories were sampled from each recording, one at a time, then increasing in length gaps were introduced in a given sampled trajectory. A matching step was performed between all trajectories and detections in the preceding frame with the detections in the frame following the gap. Number of correct matchings for the sampled trajectories with a gap were counted. Source data are provided as a Source Data file.

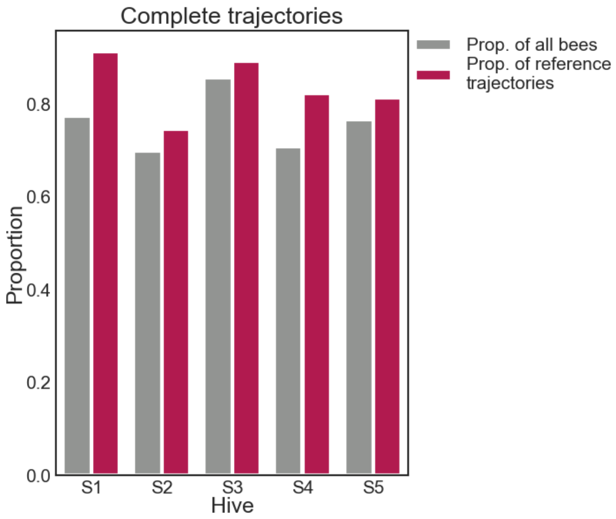

Supplementary Figure 20. Cross-validation test of the tracking accuracy. Each of the recordings was left out from the training set, then the network was trained on the validated reference trajectories of the four remaining recordings and used to calculate the embeddings of the detections in the left-out recording. The trajectories in this video were next constructed. Shown are proportions of the complete trajectories relative to the average total number of bees in the hive (gray) and relative to the number of reference trajectories (red). In this test between 70% (S2) and 86% (S3) of the detected number of bees are correctly tracked. Source data are provided as a Source Data file.

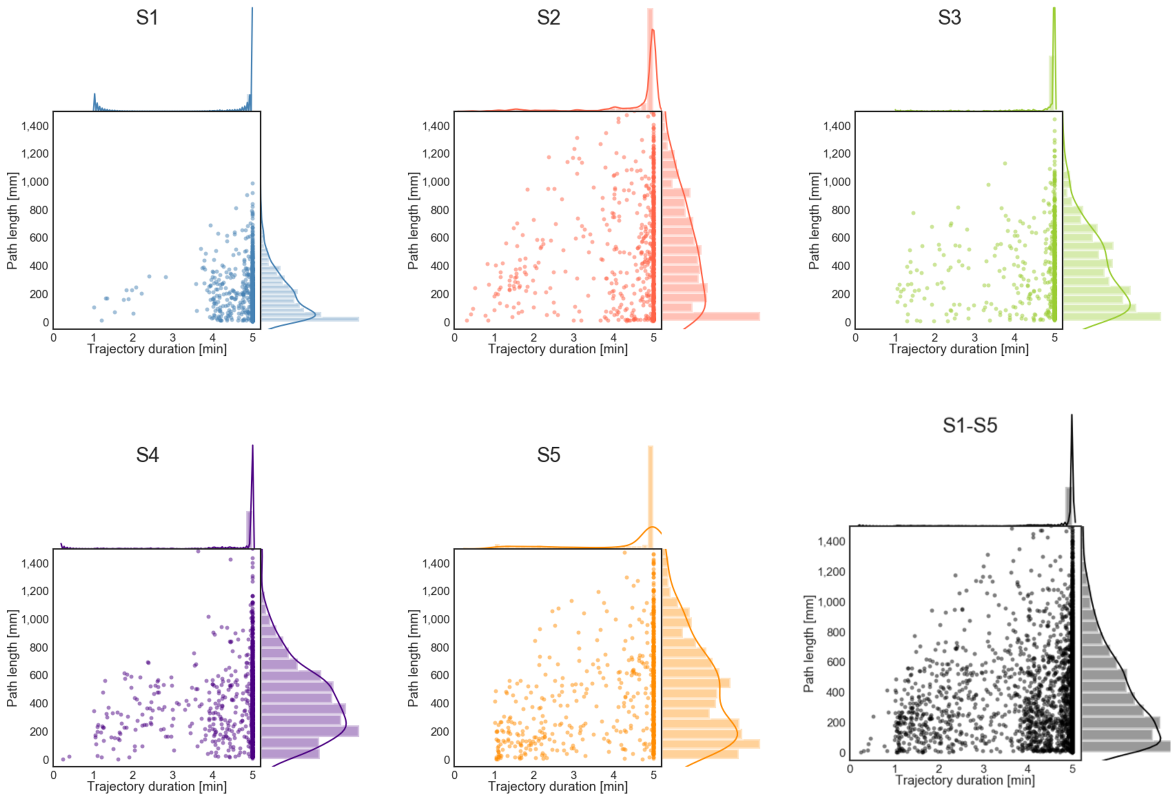

Supplementary Figure 21. Joint distribution of trajectory duration and path lengths. The path length is quantified as the sum of the distances travelled in timesteps of 1 sec. Source data are provided as a Source Data file.

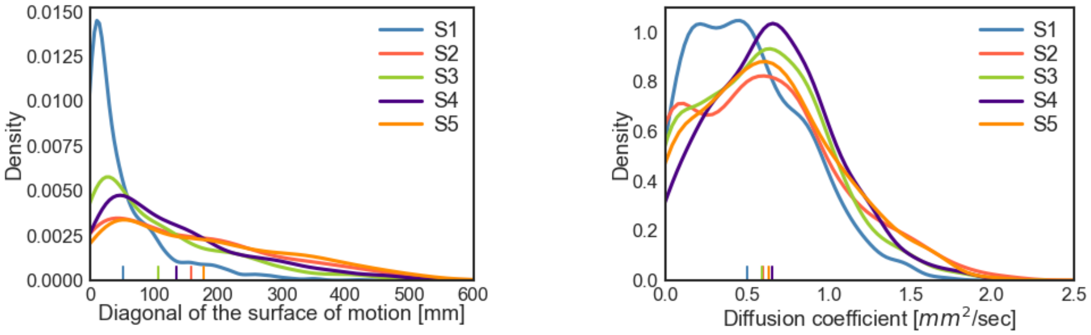

Supplementary Figure 22. Analogous to Fig. 5A-B, distributions of trajectory surface (left) and diffusion coefficient (right) in hives S1-S5. While differences in motion across the hives is observable based on the trajectory surface, mean diffusivity of the hives is comparable. The vertical lines indicate the mean value of each distribution. Source data are provided as a Source Data file.

2. Tables

Supplementary Table 1. Accuracy of detection. “Class” column indicates the average proportion of detections that were incorrectly assigned its class label: full-bees and cell-bees. “Orientation angle” refers to the error in the head-tail orientation angle. Human labelling accuracy, displayed in the first row, was assessed based on repeated annotation of four video frames by 10 independent labelers. Second row refers to the method previously published in conference proceedings (citation 18 in the main text). Last row presents results that we quantified in images of L5 and S5 that were not part of the training set. Values represent mean across video frames and standard deviation in brackets.

	**TPR**	**FPR**	**Error:**		
			**Class**	**Position [pixel]**	**Orientation angle [°]**
**Human labeling**	0.99	0.07	0.04	6.7	7.7
**Bozek et al. detection method**	0.96	0.06	0.19	5.1	9.7
**L5 and S5 recordings**	0.99 (0.005)	0.03 (0.001)	0.003 (0.06)	0.5 (2.9)	1.4 (12.7)

Supplementary Table 2. List of recordings. Beehives were imaged in two different locations with varying camera resolutions. All imaging data in this study was collected in 2018.

**Recording**	**Location**	**Start date and time**	**Pixel resolution**	**Time resolution**	**Number of frames**
L1	2	23-04 20:55	2560 x 2560	1 / 2 min	9,999
L2	2	15-05 13:00	2560 x 2560	1 / 2 min	15,124
L3	2	09-07 14:30	2560 x 2560	1 / 2 min	22,892
L4	2	31-09 12:00	2560 x 2560	1 / 2 min	12,946
L5	2	26-10 17:00	3840×2160	1 / 1 min	227,131
S1	1	12-12 09:40	5120x5120	30 fps	9,000
S2	1	15-05 10:00	5120x5120	30 fps	9,000
S3	2	27-11 10:00	3840×2160	30 fps	9,000
S4	2	25-02 11:30	3840×2160	30 fps	9,000
S5	2	19-09 09:30	3840×2160	30 fps	9,000

Supplementary Table 3. Completeness of the tracking results. The table lists the number of correct trajectories obtained with the presented methods in recordings S1-S5. Column 'mean bee count' lists the number of detections averaged across all video frames in the recording. 'Reference trajectories' lists the total number of trajectories that were collected and validated as correct for the respective beehives. The trajectories constructed by each tracking method are matched against these reference trajectories. Following columns list the raw number of correct trajectories found by each method and the same number as a proportion to the mean number of detections in parentheses. Results of the best performing method are marked in red.

Recording	Mean bee count	Reference trajectories	Position only	Pixel personality	Initial d-set	Final d-set	Data augmentation (final d-set)	Background masking (final d-set)
S1	1315.6	1115	916 (0.696)	758 (0.576)	1010 (0.768)	1046 (0.795)	1014 (0.771)	1004 (0.763)
S2	917.4	859	408 (0.445)	223 (0.243)	571 (0.622)	714 (0.778)	705 (0.769)	642 (0.700)
S3	839.1	806	598 (0.713)	523 (0.623)	700 (0.834)	724 (0.863)	722 (0.860)	687 (0.819)
S4	1278.3	1100	620 (0.485)	507 (0.397)	848 (0.663)	944 (0.738)	896 (0.701)	779 (0.609)
S5	805.5	758	275 (0.341)	-	-	617 (0.766)	556 (0.690)	381 (0.473)
